# slurmR: A lightweight wrapper for HPC with Slurm

**DOI:** 10.21105/joss.01493

**Published:** 2019-10-08

**Authors:** George G Vega Yon, Paul Marjoram

**Affiliations:** 1 Department of Preventive Medicine, University of Southern California

## Summary

Nowadays, high-performance-computing (HPC) clusters are commonly available tools for either **in** or **out** of cloud settings. Slurm Workload Manager (see Yoo et al. (2003)) is a program written in C that is used to efficiently manage resources in HPC clusters.

While the R programming language (R Core Team, 2019) has not been developed for HPC settings, there are currently several ways in which R can be enhanced by means of HPC. The slurmR R package is one of those ways.

The slurmR R package provides tools for using R in HPC settings that work with Slurm. It provides wrappers and auxiliary functions that allow the user to seamlessly integrate their analysis pipeline with HPC, putting emphasis on providing the user with a family of functions similar to those that the parallel R package (R Core Team, 2019) provides.

While there are other tools for integrating R in a HPC envirnment that works with Slurm–see for example rslurm(Marchand et al., 2017), batchtools (Lang et al., 2017), drake (Landau, 2018), future.batchtools (Bengtsson, 2019), clustermq (Schubert, 2019)–slurmR has some advantages regarding syntax, number of dependencies, and flexibility (in terms of the integration with Slurm itself). In particular, you may want to use slurmR if you:

1. Need a dependency-free tool. Besides of Slurm itself^1^, this R package only depends on other R packages that are part of base R.,
2. Need an R package that is fully integrated with Slurm, e.g., submitting jobs with an arbitrary set of Slurm parameters without the need of using templates, call Slurm commands from within R like sacct, scancel, squeue, sbatch, etc. with their corresponding flags, and
3. Want to use an R package that is ready-to-go. Once loaded, users can submit jobs by just specifying how many cores, for example, they need.

Other features that are included with this R package, and that are available in some others, are:

- It uses a syntax similar to the apply family of functions in the parallel R package, including Slurm_lapply, Slurm_sapply, Slurm_EvalQ, and Slurm_Map,
- It can resubmit failed jobs: A very common issue with heterogenous computing clusters is the fact that some jobs succeed while others fail. Partial-job-resubmission is out-of-the-box as users can specify which jobs (as in Job Arrays) should be re-run.

Both of the latter two features also available in batchtools. A comparison table of R packages that work with Slurm is available at https://github.com/USCbiostats/slurmR.

In summary, slurmR provides a dependency-free and purpose-built alternative for R users working in a HPC environment with Slurm.

## Footnotes

1 In fact, users can install this R package regardless of whether or not they have Slurm on their systems. The debug mode of this software allows users to setup jobs (including R scripts and batch files) without having to submit them to a Slurm job-scheduler.
